# Supplementary material for: Interference with DGAT Gene Inhibited TAG Accumulation and Lipid Droplet Synthesis in Bovine Preadipocytes
Source: Animals (Basel). 2023 Jul 6;13(13):2223. doi: 10.3390/ani13132223 (PMC10340012; doi:10.3390/ani13132223)
Supplement: Supplementary file 1 [file animals-13-02223-s001.zip › animals-2465666-supplementary.pdf]

Interference with DGAT Gene-Inhibited TAG Accumulation and Lipid Droplet Synthesis in Bovine Preadipocytes

Pan-Pan Guo, Xue-Rui Yao, Xin Jin, Yong-Nan Xv, Jun-Fang Zhang, Qiang Li , Chang-Guo Yan, Nam-Hyung Kim and Xiang-Zi Li

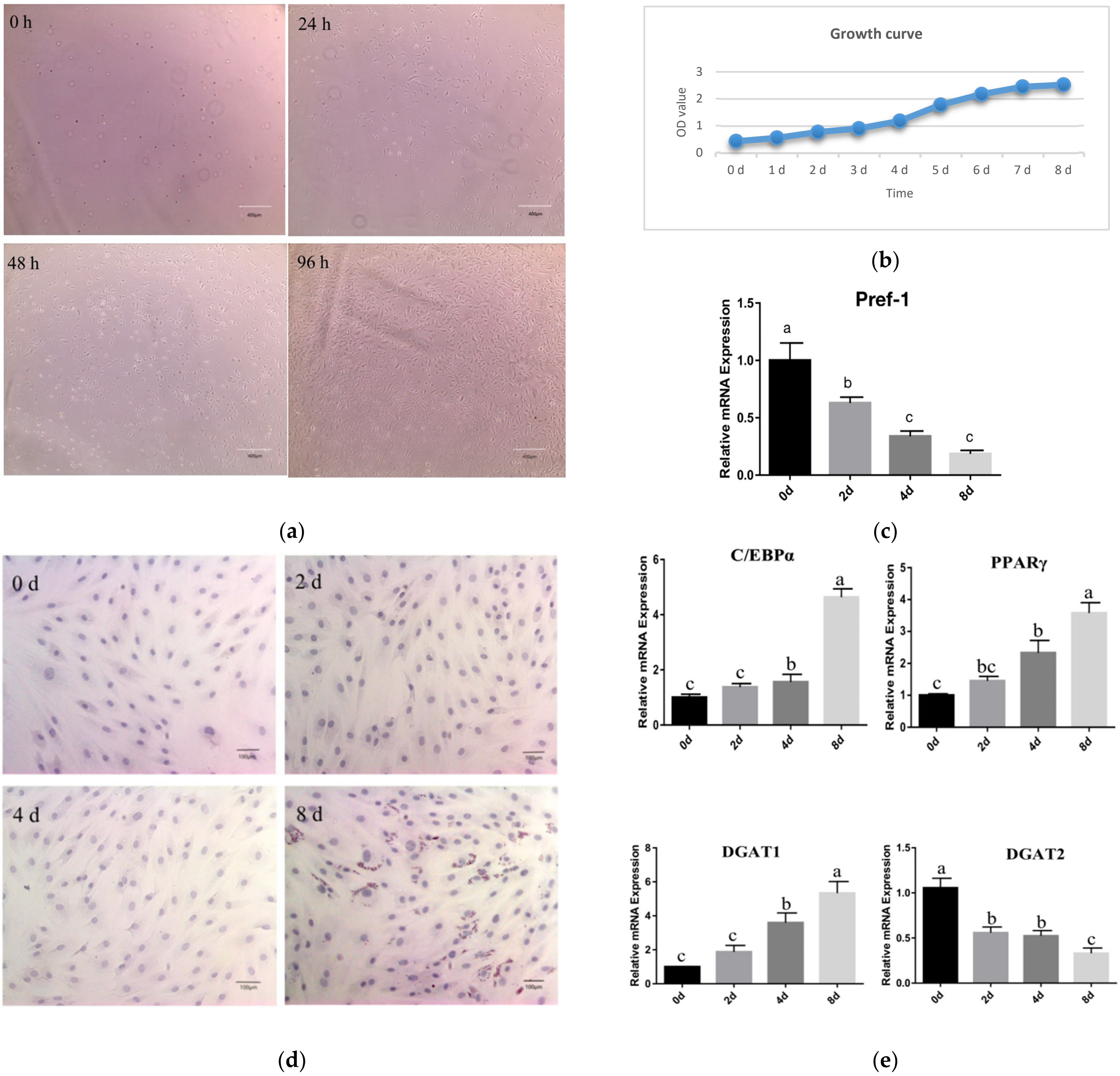

Figure S1. Isolation, culture, identification, and timing expression of DGAT gene in bovine adipocytes. **(a)** Morphology of cells (Scale bars: 400 μm). The newly inoculated cells were reduced into small spherical shapes (0 h). After 24 h, part of the cells with irregular triangles had attached to the wall (24 h), and then the attached cells gradually began to spread and

deform into uniform and complete single-compartment adipocytes and presented a long and narrow spindle fibroblast-like cell shape (48 h). The proliferation rate of the cells in continued culture was relatively slow, and the cell proliferation reached more than 80%, ready to enter the differentiation stage (96 h). **(b)** The cells had stable growth characteristics. From 2 to 7 days, the cell vitality was significantly enhanced and entered the exponential growth phase. After 7 to 8 days, entering the plateau phase, the cell proliferation rate slowed down significantly, and then gradually withdrew from the cell proliferation cycle. **(c)** Oil red O staining (Scale bars: 100 μm) and **(d-e)** time sequence expression of precursor adipocytes, lipid marker genes, and *DGAT* genes. The results were presented as the mean ± SEM (n = 3). The different letters (a–e) represent significant differences ($p < 0.05$) in gene expression.

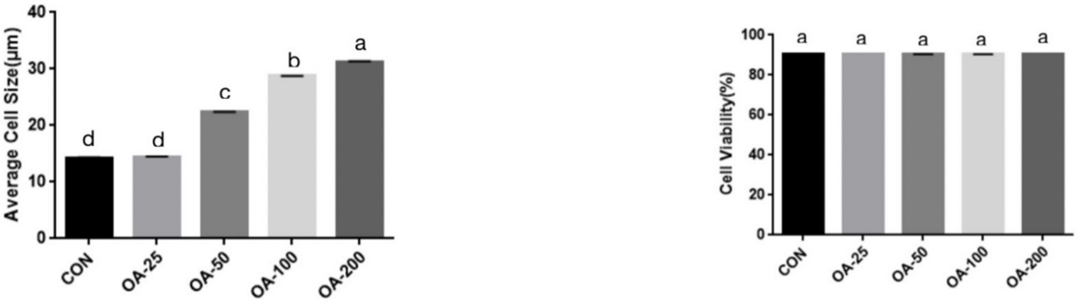

(**a**)

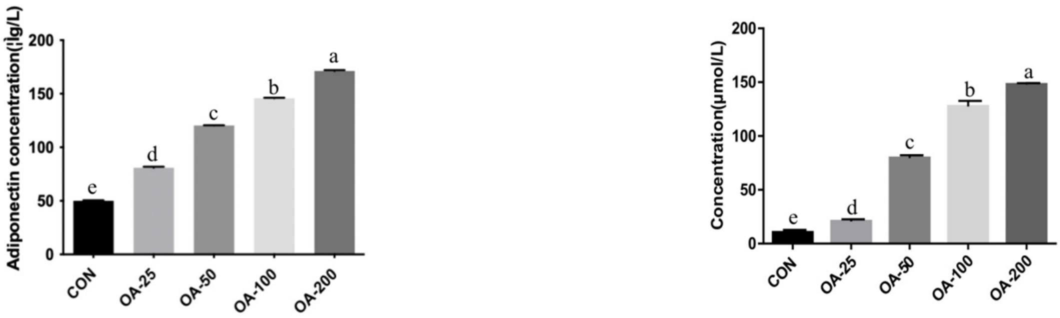

(**b**)

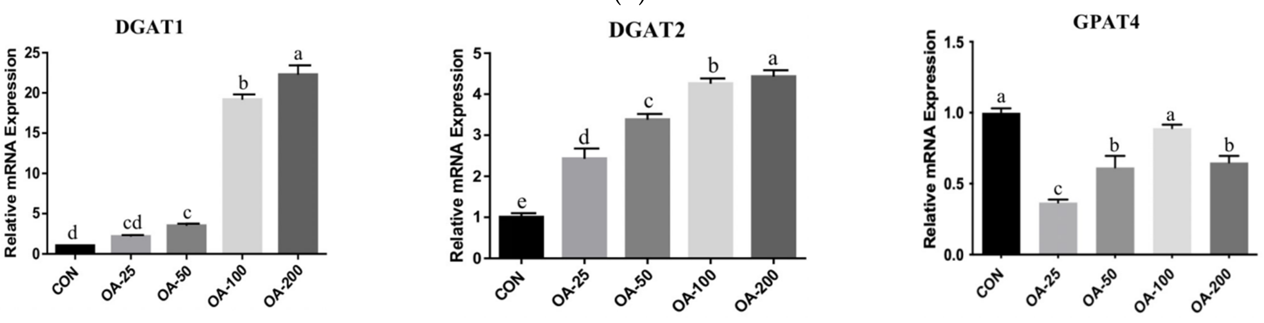

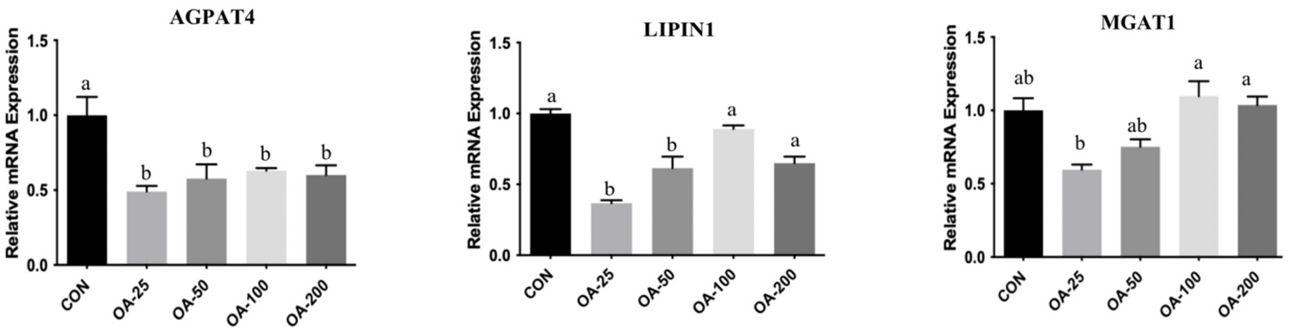

(**c**)

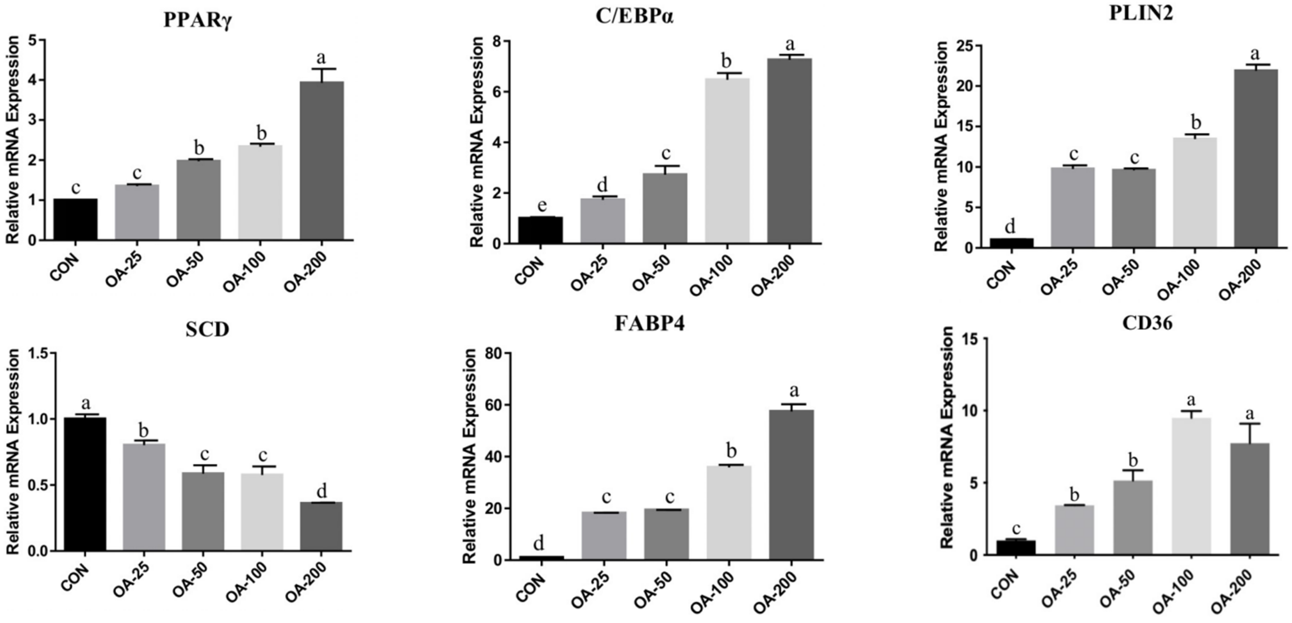

(**d**)

Figure S2. Differentiation of bovine preadipocytes induced by different concentrations of oleic acid. (a) Cell size and cell viability. (b) Concentration of triglycerides and adiponectin. (c) Expression of genes related to triglyceride synthesis pathway. (d) Expression of genes related to lipid metabolism. The results were presented as the mean ± SEM (n = 3). The different letters (a–e) represent significant differences ($p < 0.05$) in gene expression.

Table S1. Information of clean data.

Sample	Trimmed_Read_Number	Trimmed_Bases	Useful_read%	Useful_bases%
sh-1-1	36366626	5454993900	91.06	91.06
sh-1-2	40975778	6146366700	91.31	91.31
sh-1-3	42154940	6323241000	92.56	92.56
sh-NC-1	38316996	5747549400	93.13	93.13
sh-NC-2	35927164	5389074600	92.61	92.61
sh-NC-3	36048608	5407291200	92.93	92.93
sh-2-1	44365810	6654871500	91.75	91.75
sh-2-2	38107086	5716062900	92.88	92.88
sh-2-3	43070816	6460622400	92.32	92.32
sh-1+2-1	40170616	6025592400	92.99	92.99
sh-1+2-2	43323888	6498583200	92.47	92.47
sh-1+2-3	40149380	6022407000	91.74	91.74

Table S2. Summary of sequencing data and reference genome comparison.

Sample	Clean Reads	TotalMapped	Multiple Mapped	Uniquely Mapped	Map Events	Mapped to Gene	Mapped to Inter Gene	Mapped to Exon
sh-NC-1	44026548	42389681 (96.28%)	1002846 (2.37%)	41386835 (97.63%)	41386835	36471282 (88.12%)	4915553 (11.88%)	33540110 (91.96%)
sh-NC-2	42622942	40922553 (96.01%)	1120749 (2.74%)	39801804 (97.26%)	39801804	34979444 (87.88%)	4822360 (12.12%)	32691531 (93.46%)
sh-NC-3	43119516	41501907 (96.25%)	1093019 (2.63%)	40408888 (97.37%)	40408888	35378530 (87.55%)	5030358 (12.45%)	32742709 (92.55%)
sh-1-1	36366626	35017719 (96.29%)	926080 (2.64%)	34091639 (97.36%)	34091639	30616505 (89.81%)	3475134 (10.19%)	28646689 (93.57%)
sh-1-2	40975778	39468963 (96.32%)	1049040 (2.66%)	38419923 (97.34%)	38419923	34574231 (89.99%)	3845692 (10.01%)	32528805 (94.08%)
sh-1-3	42154940	40498526 (96.07%)	1138229 (2.81%)	39360297 (97.19%)	39360297	34520961 (87.71%)	4839336 (12.29%)	31736725 (91.93%)
sh-2-1	44365810	42604283 (96.03%)	1106723 (2.60%)	41497560 (97.40%)	41497560	37195112 (89.63%)	4302448 (10.37%)	34794332 (93.55%)
sh-2-2	38107086	36636777 (96.14%)	936978 (2.56%)	35699799 (97.44%)	35699799	32098875 (89.91%)	3600924 (10.09%)	30060957 (93.65%)
sh-2-3	43070816	41277842 (95.84%)	1181981 (2.86%)	40095861 (97.14%)	40095861	34812914 (86.82%)	5282947 (13.18%)	32063210 (92.10%)
sh-1+2-1	40170616	38605876 (96.10%)	1007014 (2.61%)	37598862 (97.39%)	37598862	33730066 (89.71%)	3868796 (10.29%)	31599534 (93.68%)
sh-1+2-2	43323888	41295601 (95.32%)	1140781 (2.76%)	40154820 (97.24%)	40154820	35535525 (88.50%)	4619295 (11.50%)	33119936 (93.20%)
sh-1+2-3	40149380	38497340 (95.89%)	1120920 (2.91%)	37376420 (97.09%)	37376420	32347092 (86.54%)	5029328 (13.46%)	30001243 (92.75%)

Table S3. Sequence information of PCR primers

Gene	Sense Strand (5'→3')	Length (bp)	Gene ID
GAPDH	F-ACTCTGGCAAAGTGGATGTTGTC<br>R-GCATCACCCCACTTGATGTTG	143	NM_001034034

FABP3	F-GAGACCACAGCAGATGACAGGAAAG<br>R-CGTCAACCATCTCCCGCACAAG	118	NM_174313.2
OLR1	F-CTTGTCTTTGGATGCCCAGT<br>R-ATTGACAACCCCATCCAGAA	108	NM_174132.2
ACSL1	F-TCCACAAGGGCTTCAAGGCA<br>R-TGGCTTCAGTTCCGAGGGTG	144	NM_001076085.1
ACOX2	F-GGAAAGCCAGCCAGCGATTC<br>R-AGCCGTCATGGAGGAAGTCG	101	NM_001102015.2
PIK3CD	F-TCCTGGACAAGATCCTGGAG<br>R-AGGTCCTTCTCGTGCTCGTA	130	NM_001205548.2
STRADB	F-CCTCATTTCTGGTGATGGCCTAGTG<br>R-CCTATGCCTCTGTCCGTGCTTAAC	82	NM_001192081.2
FBP1	F-AGAAGGCAGGAGGAATGGCTACC<br>R-GATGATGGGCGACTTCTGATGGATG	86	NM_001034447.2
CFTR	F-GAGGCAGTCCGTTCTGAACCTTATG<br>R-TTCGTGTGGATGTCGCTGTCTTTC	86	NM_174018.2
SFRP2	F-TCCTGGAGACCAAGAGCAAGACC<br>R-GAGCCACAGCACCGACTTCTTC	80	NM_001034393.1
MAPK10	F-GACCACGAGCGAATGTCTTACCTG<br>R-CCCTGTGAATAATCCCAGCAGAGTG	82	NM_001083728.3
SFRP4	F-AACGACAGTGGTGGATGTGAAAGAG<br>R-CTTGATGAGGCAGGATGTGTGGAC	116	NM_001075764.1
WNT2	F-CTGTCTCGTTGGAATCTGGCTCTG<br>R-TGTCACACATCACCCTGGAGGAG	119	NM_001013001.1
